# Peer Review of “Forecasting the COVID-19 Pandemic in Saudi Arabia Using a Modified Singular Spectrum Analysis Approach: Model Development and Data Analysis”

**DOI:** 10.2196/28679

**Published:** 2021-03-31

**Authors:** Anonymous Reviewer

**Keywords:** COVID-19, prediction, singular spectrum analysis, separability, eigenvalues, Saudi Arabia

*This is a peer review submitted for the paper "Forecasting the COVID-19 Pandemic in Saudi Arabia Using a Modified Singular Spectrum Analysis Approach: Model Development and Data Analysis".*

## Round 1 Review

Dear Author,

Thank you for the opportunity to review your paper [1].

I believe your manuscript would benefit from an editorial review prior to resubmission. This should include several elements: semantic and syntax review, native speaker edits, and formatting. Some of the words are illegible: modified presents as “modi ed”, different reads “di erent”, and many more. There are many words that appear incomplete or fragmented, which generally renders the manuscript illegible. This may have been due to a formatting bug during submission.

At this point, the paper should be reworked and then resubmitted so that an appropriate content review can take place.

Thank you and best.

## Round 2 Review

Dear Author,

Thank you for addressing the formatting issue in your manuscript. However, I believe your manuscript would still benefit from an editorial review with respect to language prior to resubmission. This should include several elements: semantic review, syntax review, and native speaker edits (words such as “glop”). Also, I suggest removing references from the Abstract and adding them to the Introduction section of your paper.

Otherwise, I believe your paper will add an interesting viewpoint from a statistical perspective to COVID-19 modeling.

Thank you and best.
